# Choledochal cysts type VI: biliary cyst of the cystic duct with connecting to right anterior sectoral bile duct: a case report

**DOI:** 10.1093/jscr/rjac126

**Published:** 2022-03-30

**Authors:** Nattaporn Maneepairoj, Pipit Burasakarn, Anuparp Thienhiran, Pusit Fuengfoo, Sutdhachit Linananda, Sermsak Hongjinda

**Affiliations:** Division of HPB Surgery, Department of Surgery, Phramongkutklao Hospital, Thung Phaya Thai, Ratchathewi, Bangkok, Thailand; Division of HPB Surgery, Department of Surgery, Phramongkutklao Hospital, Thung Phaya Thai, Ratchathewi, Bangkok, Thailand; Division of HPB Surgery, Department of Surgery, Phramongkutklao Hospital, Thung Phaya Thai, Ratchathewi, Bangkok, Thailand; Division of HPB Surgery, Department of Surgery, Phramongkutklao Hospital, Thung Phaya Thai, Ratchathewi, Bangkok, Thailand; Division of HPB Surgery, Department of Surgery, Phramongkutklao Hospital, Thung Phaya Thai, Ratchathewi, Bangkok, Thailand; Division of HPB Surgery, Department of Surgery, Phramongkutklao Hospital, Thung Phaya Thai, Ratchathewi, Bangkok, Thailand

## Abstract

Type VI choledochal cysts or cystic duct dilatation cysts are a relatively new and rare condition. We report the case of a 35-year-old man who presented with a history of recurrent episodes of epigastrium pain. Magnetic resonance cholangiography revealed a cyst lodged between the cystic duct and the right anterior sectoral bile duct. He underwent a laparoscopic right anterior sectorectomy with cholecystectomy. Pathological examination revealed a cyst with a fibrous wall, dense chronic inflammatory infiltration, lined by columnar epithelium. Due to its rarity, the diagnosis is often made intraoperatively. The treatment of cystic duct cysts includes cholecystectomy, complete cyst excision, recontinuity of the common bile duct. Type VI choledochal cysts are extremely rare. Preoperative diagnosis, using either magnetic resonance cholangiopancreatography or endoscopic retrograde cholangiopancreatography, is vital to prevent postoperative complications. Treatment of this type of cysts includes cholecystectomy and complete cyst excision and biliary-enteric reconstruction if necessary.

## INTRODUCTION

Choledochal cysts are rare congenital anomalies that involve the dilatation of the extrahepatic or intrahepatic bile duct. Out of the five conventional types of choledochal cysts [1], a novel type, type VI choledochal cysts or cystic duct dilatation cysts, is a relatively new and rare condition [2], with only <50 reported cases. The treatment of choledochal cysts involves the complete excision of the cyst, with or without hepatectomy and recontinuation of the bile duct with biliary-enteric reconstruction. We report the case of a 35-year-old man who presented with epigastric pain and a type VI choledochal cyst.

## PRESENTATION OF A CASE

**Figure 1:**
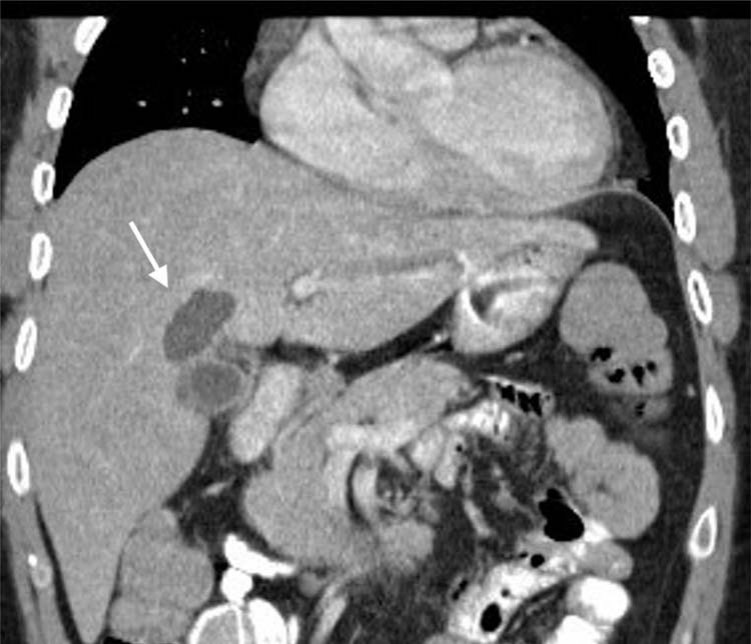
Computed tomography image showing an intrahepatic cyst (arrow).

**Figure 2:**
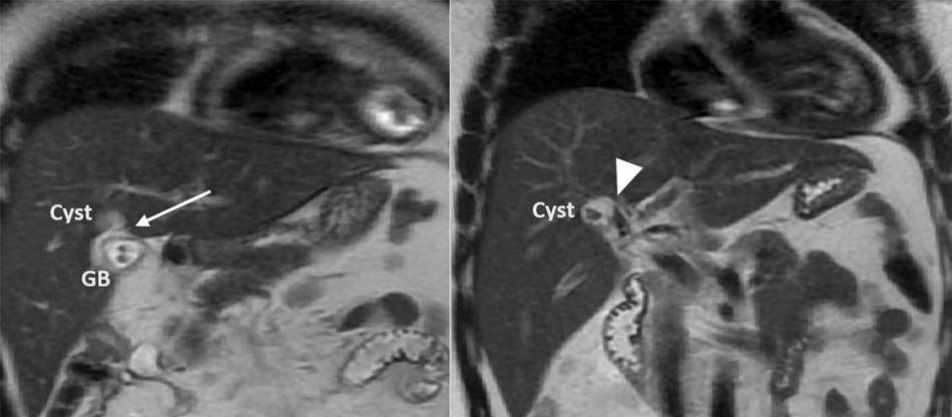
MRCP showing a cyst connected to the cystic duct (arrow) and the right anterior sectoral bile duct (arrowhead).

**Figure 3:**
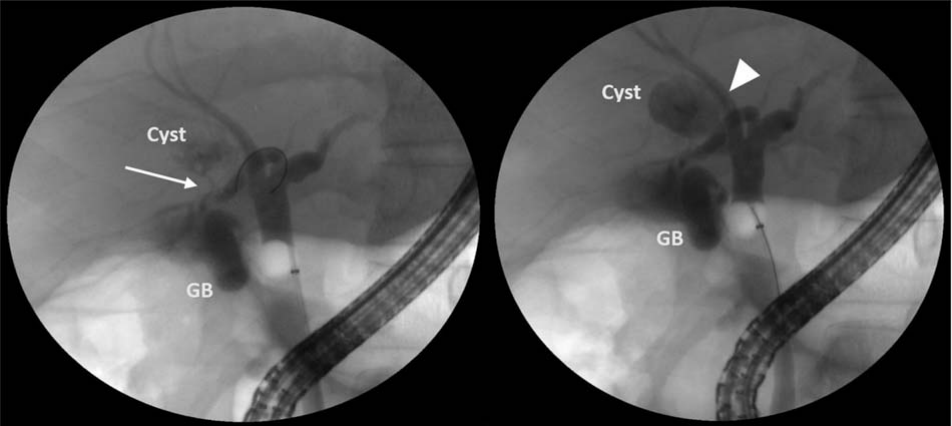
ERCP showing that the cyst can be filled with contrast from the cystic duct (arrow) and the right anterior sectoral bile duct (arrowhead).

**Figure 4:**
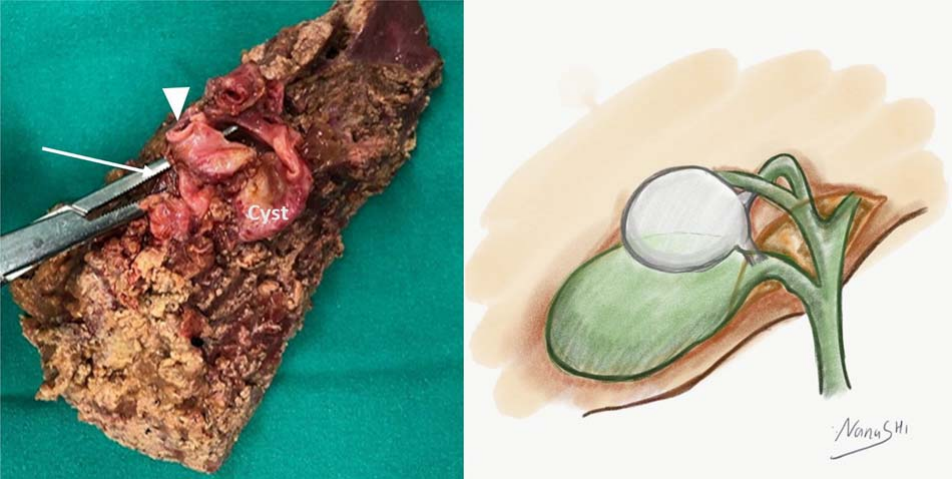
[Left] the surgical specimen revealed that the lumen of the cyst was connected to the right anterior sectoral bile duct (arrow) and the cystic duct (arrowhead). [right] Diagram of the cystic duct cyst.

**Figure 5:**
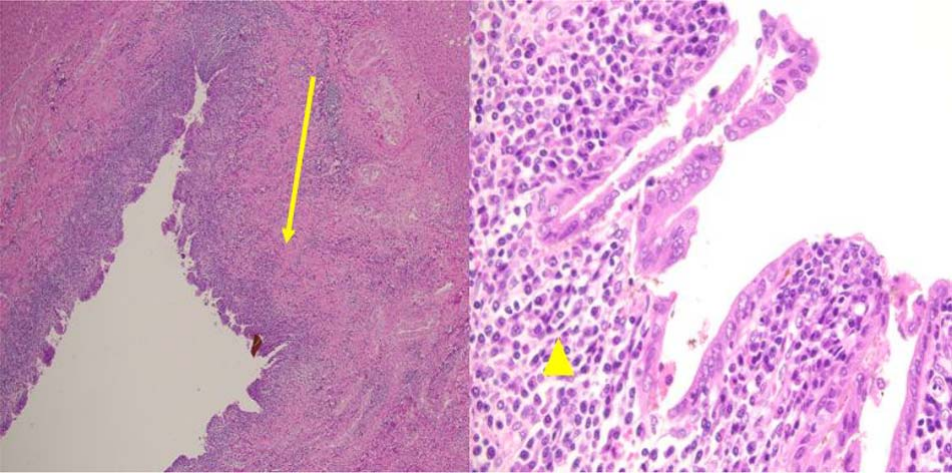
Pathological examination revealed a cyst with a fibrous wall (arrow), dense chronic inflammatory infiltration, lined by columnar epithelium (arrowhead). Mild reactive atypia, associated with marked chronic inflammatory cell infiltration, was noted. No signs of epithelial dysplasia were observed.

A 35-year-old man presented with a history of recurrent episodes of epigastric pain. Ultrasonography revealed an intrahepatic cyst (Fig. 1), and magnetic resonance cholangiography revealed that the cyst was connected to the cystic duct and right anterior sectoral bile duct (Fig. 2). The patient underwent an endoscopic retrograde cholangiography, which showed that the contrast could fill the cyst (Fig. 3). The laboratory findings, including a complete blood count, showed a white blood cell count of 4200/mm^3^ (lymphocyte 23%, neutrophil 62%); haemoglobin level of 16 g/dL; platelet count of 215 × 103/ mm^3^; total protein of 7.8 g/dL; albumin of 4.3 g/dL; total bilirubin level of 0.68 mg/dL; serum aspartate aminotransferase level of 26 IU/L; serum alanine aminotransferase level of 29 IU/L and alkaline phosphatase level of 105 IU/L. Tumour markers, including alpha-fetoprotein, carcinoembryonic antigen and carbohydrate antigen 19–9 were within normal limits. Viral markers for hepatitis B and C were negative. Due to the preoperative diagnosis of a type VI choledochal cyst, with a differential diagnosis of cystic intraductal papillary neoplasm of the bile duct, the patient underwent laparoscopic right anterior sectorectomy to excise the cyst completely. During the operation, after identifying the cystic duct and taking down the fundus and body of the gallbladder from the liver bed, an intraoperative cholangiogram was performed via the cystic duct connected to the cyst. Right anterior sectorectomy was then performed (Fig. 4). The postoperative period was unremarkable, and the patient was discharged on the sixth postoperative day. Pathological examination revealed a cyst with a fibrous wall, dense chronic inflammatory infiltration, lined by columnar epithelium. Mild reactive atypia, associated with marked chronic inflammatory cell infiltration, was noted. No signs of epithelial dysplasia were observed (Fig. 5).

## DISCUSSION

Type VI choledochal cysts were first described by Bode and Aust [3] in a 7-year-old Mexican-American girl who presented with jaundice. At the time of consultation, a dilated cystic duct cyst with compression of the common bile duct was noted. The patient underwent cholecystectomy with complete cyst excision and choledochoduodenostomy. Serena *et al*. included cystic duct cysts into the new type VI choledochal cyst classification [2]. Previous analysis of world literature [4] revealed slight predominance of type VI choledochal cyst in females and the 18–55 years age group. The pathogenesis of type VI choledochal cysts is still debated; no definitive theory has been proven. The abnormal pancreatobiliary ductal junction (APBJ) [5] is commonly accepted and accounts for up to 40% of the observed cases. This anomaly is associated with a long common channel that predisposes patients to reflux of pancreatic juice into the biliary tree, leading to inflammation, ectasia and dilation. A morphology of this type can also be associated with fusiform (more common) or saccular cyst dilatation [6].

The clinical symptoms of patients with this type of cyst are often similar to those with other types of choledochal cysts, which vary from asymptomatic to right upper quadrant pain, jaundice or cholangitis, depending on the size and the pressure effect created by the cyst [7]. Due to its rarity, the diagnosis is often made intraoperatively [8]. However, an accurate preoperative diagnosis is essential to prevent surgical complications. Thus, preoperative imaging studies, such as magnetic resonance cholangiopancreatography (MRCP) and endoscopic retrograde cholangiopancreatography (ERCP), may be used for preoperative diagnosis [9]. MRCP helps detect the location of the cyst and its relationships, the presence of possible APBJ and other complications. However, ERCP, which is the gold standard for diagnosis but is invasive and leads to post-procedure complications, indicates that MRCP cannot provide useful information on when a therapeutic procedure is required. Treatment of type VI choledochal cyst depends on its morphology guided by preoperative imaging studies. Complete cyst excision and cholecystectomy are usually sufficient for cases with a narrow connection between the cyst and the common bile duct; however, in cases with a wide connection between the cyst and the common bile duct, biliary-enteric reconstruction (hepaticojejunostomy) may require cyst excision and cholecystectomy [6, 8]. In our case, cholecystectomy and right anterior sectorectomy were performed laparoscopically because of the narrow connection between the cystic duct cyst and the right anterior sectoral bile duct. The patient was discharged without postoperative complications.

## CONCLUSION

Cystic duct cysts or type VI choledochal cysts are extremely rare. Preoperative diagnosis, using either MRCP or ERCP, is vital to prevent postoperative complications. Treatment of this type of cysts includes cholecystectomy and complete cyst excision and biliary-enteric reconstruction if necessary.

## DECLARATION OF FIGURES’ AUTHENTICITY

All figures submitted have been created by the authors, who confirm that the images are original with no duplication and have not been previously published in whole or in part.
